# Vagus nerve stimulation lead removal or replacement: surgical technique, institutional experience, and literature overview

**DOI:** 10.1007/s00701-015-2547-9

**Published:** 2015-09-03

**Authors:** Marlien W. Aalbers, Kim Rijkers, Sylvia Klinkenberg, Marian Majoie, Erwin M. J. Cornips

**Affiliations:** Department of Neurosurgery, University Medical Center Groningen, Hanzeplein 1, 9700 RB Groningen, The Netherlands; Department of Neurosurgery, Atrium-Orbis Heerlen, PO Box 4446, 6401 CX Heerlen, The Netherlands; Department of Neurology, Maastricht University Medical Center, PO Box 5800, 6202 AZ Maastricht, The Netherlands; Department of Neurosurgery, Maastricht University Medical Center, PO Box 5800, 6202 AZ Maastricht, The Netherlands; Epilepsy Center Kempenhaeghe, PO Box 61, 5590 AB Heeze, The Netherlands

**Keywords:** Vagus nerve stimulation, Lead, Revision, Epilepsy, Neuromodulation

## Abstract

**Background:**

With the growing use of vagus nerve stimulation (VNS) as a treatment for refractory epilepsy, there is a growing demand for complete removal or replacement of the VNS system. We evaluate the safety and efficacy of complete removal or replacement of the VNS system and provide an extensive description of our surgical technique.

**Methods:**

We retrospectively reviewed our patient registry for all VNS surgeries performed between January 2007 (the year of our first complete removal) and May 2014. In order to assess patient satisfaction, a written questionnaire was sent to patients or their caregivers. Additionally, we reviewed all literature on this topic.

**Results:**

The VNS system was completely removed in 22 patients and completely replaced in 13 patients. There were no incomplete removals. Revision surgery was complicated by a small laceration of the jugular vein in two patients and by vocal cord paralysis in one patient. Seizure frequency was unaltered or improved after revision surgery. Electrode-related side effects all improved after revision surgery. Twenty-one studies reported a total of 131 patients in whom the VNS system was completely removed. In 95 patients, the system was subsequently replaced. The most frequently reported side effect was vocal cord paresis, which occurred in four patients.

**Conclusions:**

Complete removal or replacement of the VNS system including lead and coils is feasible and safe. Although initial results seem promising, further research and longer follow-up are needed to assess whether lead replacement may affect VNS effectiveness.

**Electronic supplementary material:**

The online version of this article (doi:10.1007/s00701-015-2547-9) contains supplementary material, which is available to authorized users.

## Introduction

Vagus nerve stimulation (VNS) is a neuromodulatory treatment that consists of chronic intermittent electrical stimulation of the left vagus nerve, delivered by a programmable pulse generator. This pulse generator is implanted subcutaneously in the chest wall and connected to a bipolar lead with three helical coils (two stimulation electrodes and an anchoring tether) wrapped around the cervical part of the vagus nerve. VNS is used as an adjunctive treatment for patients with refractory epilepsy who are not eligible for resective surgery or in whom resective surgery has failed. The treatment is generally well tolerated and severe side effects are rare [4, 13, 15, 26]. Additionally, VNS is approved for refractory depression, while other indications are being investigated including advanced heart failure [3, 20, 21].

**Fig. 1:**
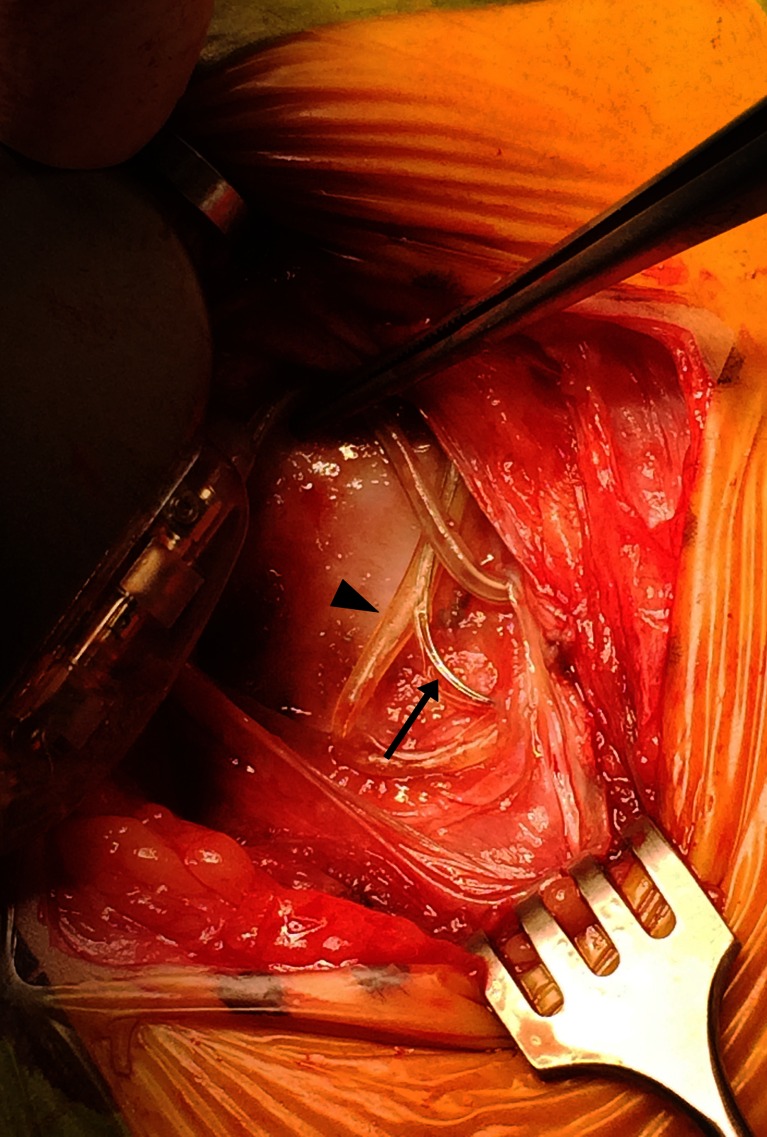
Intraoperative image: once the generator is moved out of its pocket, degradation of the silicone coating of the lead is clearly visible. Note the exact point where the lead has left the silicone coating (arrowhead) and the trajectory it has subsequently followed (arrow)

Well over 85.000 epilepsy patients have been implanted with a VNS device [7]. With a growing number of implants, a growing need for removal or replacement of the VNS system has emerged. Whereas the generator is removed or replaced routinely, the leads are considered more challenging because of postoperative scarring close to the larynx, internal jugular vein, carotid artery, and electrode-nerve complex. In case of VNS removal it has been advocated to cut the lead a few centimeters proximal to the coils and leave the rest in situ in order not to damage the nerve. However, complete removal may sometimes be necessary, for instance in case of late onset infection or hardware failure e.g. degradation of the silicone coating (Fig. 1). Furthermore, complete removal enables unrestricted use of (high-field) MR imaging techniques that may be needed in patients that are re-evaluated for epilepsy surgery [8]. Finally, some patients in whom VNS is ineffective strongly desire complete explantation of the device. The question therefore arises whether complete removal of VNS lead and coils is feasible, safe, and whether subsequent replacement will affect treatment response. In this paper we report our experience and review the current literature on VNS revision and removal. We demonstrate that complete removal or replacement of the VNS system including lead and coils is feasible and safe, and provide a detailed description of our surgical technique.

## Materials and methods

### Case selection

The Medical Ethical Committee of Maastricht University Medical Center approved this study. We obtained informed consent to access medical records for research purposes and retrospectively reviewed our patient registry for all VNS surgeries performed at Maastricht University Medical Center, a tertiary referral hospital, between January 2007 (the year of our first complete removal) and May 2014. We retrieved the following data from inpatient and outpatient records: patient sex, age at revision, interval between implantation and revision, presenting symptoms, device diagnostics, indications for removal or replacement, intraoperative findings and hardware failure, complications, and adverse events.

### Patient satisfaction

A written questionnaire was sent to all patients or caregivers, asking them whether seizure frequency and severity were “worse”, “unchanged”, or “better” compared with preoperatively and whether they were satisfied with the result of the operation. Patients were also asked whether surgery affected preoperative side effects that occurred with their previous VNS implant, and whether new adverse events or side effects occurred after surgery.

### Surgical technique (supplemental video)

**Fig. 2:**
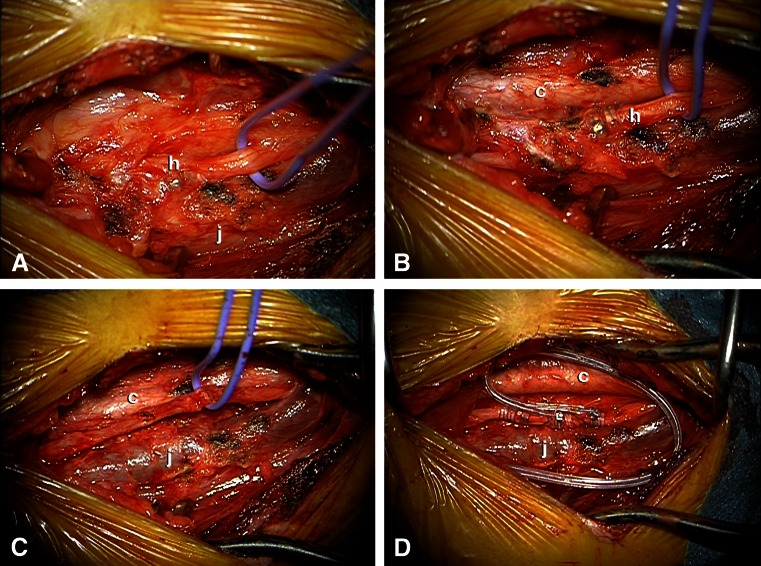
Intraoperative images before (**a**, **b**) and after (**c**) removal of the helices and finally with the new lead in place (**d**). A blue vessel loop surrounds the vagus nerve (*c*, carotid artery;* h*, helices;* j*, internal jugular vein)

**Fig. 3:**
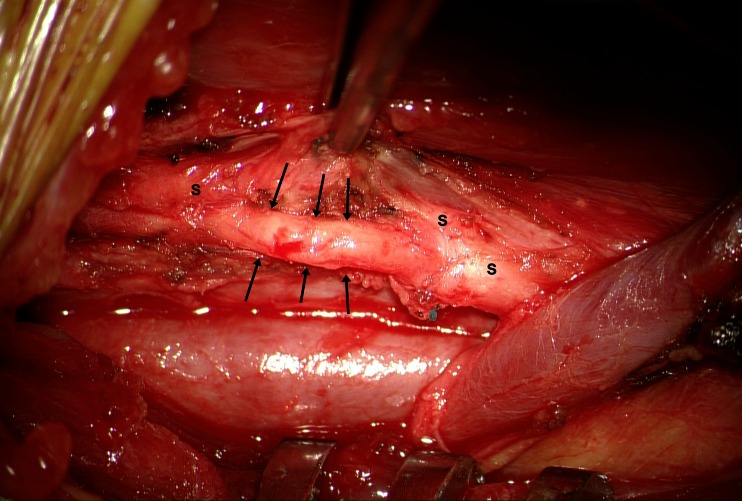
Intraoperative image demonstrating short segment atrophy of the vagus nerve as a result of chronic constriction caused by suboptimal position of the helices and/or strain relief loops. Impressions caused by three individual helices (especially the middle one) are clearly visible (*arrows*). The instrument is holding scar tissue (s) attached to and surrounding the vagus nerve

All procedures but one were performed by the same neurosurgeon (EC). The operation was performed after administration of intravenous antibiotics and under strict aseptic conditions according to our protocol for surgical implants. The generator was gently moved out of its pocket to disconnect the lead. The horizontal neck incision was reopened, exposing the lead. Both fixation booklets were removed and the lead was carefully followed through the scar tissue medial to the sternocleidomastoid muscle towards the neurovascular bundle containing the carotid, internal jugular vein, and vagus nerve. As in primary implantations, it is imperative to convert an aesthetic horizontal incision into a sufficiently large vertical exposure by mobilizing the platysma on both sides from underlying tissues and keeping it away with a Gelpi retractor positioned parallel to the medial sternocleidomastoid muscle border. The omohyoid muscle is mobilized using sharp dissection and retracted in cranial (or less frequently caudal) direction using the Gelpi retractor. With gentle longitudinal dissection (paralleling the neurovascular structures) using a small scissor with blunt, curved tips the lead is followed to the helices that may easily be palpated before being exposed. This also helps to locate the carotid that may remain attached with its medial border to the laryngeal structures. Likewise, the internal jugular vein may remain attached with its lateral border to the inner surface of the sternocleidomastoid muscle, while the nerve is exposed in between both vessels. The Gelpi is now carefully repositioned paralleling the internal jugular vein, and the operating microscope is installed (supplemental video). The helices are exposed and removed in a cranio-caudal direction. In case of lead removal, the nerve does not need to be detached from adhesions underneath, whereas in case of lead replacement, the nerve needs to be detached very carefully using scissors while it is gently lifted with a vessel loop. Excessive (cranio-caudal) traction on the nerve should be avoided at all times. We have tried microscissors to cut the coils turn by turn, however, we soon discovered that regular scissors with straight and blunt, curved tips do a much better job. Importantly, we have always observed a cleavage plane in between the nerve on the one end and the helices surrounded by multiple layers of scar tissue on the other end. This cleavage plane can be found with surprising ease when the most cranial helix of the most cranial coil is gently lifted and cut with a small scissor under microscopic view. Subsequently, the three coils are removed in a piecemeal fashion, exposing the vagus nerve, which usually looks surprisingly normal thereafter (Fig. 2), except for those rare cases where we observed a kink in the nerve caused by a suboptimal position of the helices and/or strain relief loops. In such patients, short segment atrophy of the nerve due to chronic constriction may be observed (Fig. 3). We never leave a coil behind, and prefer to position the coils of the new lead in the exact same position so as to avoid exposure and devascularization of an unnecessarily long nerve segment. Especially in case of reimplantation, the surgical field is repeatedly irrigated with saline solution containing gentamicin. Once all hardware has been completely removed, reimplanting an entirely new VNS system is as straightforward as the initial procedure, and the wound is closed in a regular fashion.

### Literature review

We performed a literature search in PubMed, Google Scholar, and Embase using the keywords “vagus nerve stimulation” or “vagal nerve stimulation”, or “VNS”, combined with “removal”, “replacement”, or “revision”. Only original studies written in English, describing removal or replacement of the VNS system including the leads, were included. Whenever it was unclear whether the lead was completely removed, the authors of the original paper were contacted to provide extra information.

## Results

### Institutional experience

Between January 2007 and July 2014, 35 patients underwent VNS removal or revision surgery, including 25 adults (mean age at surgery 34 years, range, 18–62 years) and ten children (mean age at surgery 16 years, range 11 to 17 years). All revisions (removal with or without replacement) were first revisions and the mean interval between initial implantation and revision was 7 years (range, 1–17 years).

#### VNS removal

In 19 patients, the VNS system was completely removed. In three additional patients, generator and proximal part of the lead had been removed in another hospital, leaving the distal part of the lead and helices wrapped around the nerve to be removed at our center. All attempted removals were complete, and there were no incomplete removals during the study period in our institution. The interval between lead implantation and removal was on average 7 years (range, 1–14 years). All patients requested removal because VNS was ineffective. Additionally, five patients required high-field MR imaging, two patients experienced paresthesias over a damaged lead, and one patient experienced discomfort because of a subcutaneously mobile generator. In one patient, an 11-year-old boy, full VNS replacement was planned but abandoned because the nerve appeared to be severely damaged and atrophied due to chronic constriction as a consequence of a suboptimal position of the helices and/or strain relief loops. In two patients, a small laceration of the internal jugular vein occurred, which was easily repaired with a polypropylene 6/0 suture.

#### VNS replacement

In 13 patients, the VNS system was completely removed and replaced on average 8 years (range, 3–17 years) after initial implantation. Nine patients presented with an increasing seizure frequency and/or seizure severity. In two patients, the generator was routinely replaced as the battery was near end of life, however, as we observed a breach in the silicone insulation of the lead (Fig. 1), we decided to replace the entire system. One patient presented with new unexplained side effects (cough and dyspnea) suggesting a dysfunctional lead. Although nothing abnormal was observed intraoperatively, the complaints disappeared after lead replacement. One patient presented with an obvious lead fracture. One patient complained of hoarseness and dysphonia immediately after surgery and was diagnosed with left vocal cord paralysis. At 3-year follow-up, the patient merely experienced stimulation-induced dysphonia, while during the stimulation-free interval, his voice was normal.

#### Patient satisfaction

At the time of follow-up, two patients were deceased. Sixteen of the remaining 33 patients (48 %) completed and returned the questionnaire, including 11 removals and five replacements. Regarding seizure frequency and severity, three of five patients (60 %) with a replacement reported less frequent and less severe seizures, whereas the other two (40 %) reported no obvious change. Among 11 patients in whom the VNS system was completely removed, two (18 %) reported less frequent seizures and nine (82 %) reported no obvious change, while seizure severity was unaffected in all. Those suffering from electrode-related side effects prior to removal (*n* = 3) reported no more side effects after removal.

### Literature review

We identified seven studies [1, 9, 11, 17–19, 30] and five case reports [12, 23, 27–29] focusing on lead removal or replacement. Additionally, nine studies described lead removal or replacement as part of a broader report on VNS treatment [2, 6, 10, 14, 16, 22, 24, 25, 31]. The included studies are summarized in Table 1. In 131 patients (including 52 children) the lead was completely removed and in 95 of them the lead was replaced. The interval between implantation and removal or replacement ranged between 1 month and 11 years. The main reasons for removal were lack of response and the occurrence of adverse effects, such as increased seizure frequency, unpleasant sensation, or impaired swallowing. Main reasons for replacement were device malfunction and infection. Complete removal or replacement were complicated by vocal cord paresis (*n* = 4), which resolved in three patients [19, 22, 29], but was permanent in one patient [19].

**Table 1:** Literature overview: original studies describing removal or replacement of the VNS system including the leads

Study	No. of patients	Children	Mean interval in months (range)	Old lead removed	Old lead not removed	New lead inserted	Reason (number of patients)	Presenting symptom (number of patients)	Hardware failure (number of patients)	Complications
Agarwal 2011	23	23	30 ± 17	18	5	23	Device malfunction (20) Infection (3)	Increased impedance (20)	Lead fracture (7)	0
Air 2009	8	8	n.m.	7	1	5	Infection (8)	Infected wound (8)	None	0
Ching 2013	6	0	n.m.	6	0	0	No response (3) Imaging required (1) Adverse effect (2)	n.m.	Lead fracture (2)	0
Dlouchy 2012	25	1	60 (22–133)	25	0	25	Device malfunction (20)	Increased seizure frequency (18) Adverse response (4) Increased impedance (18)	Lead fracture (3)	1 taut cable
Elliot 2011	26	n.m.	n.m.	7	19	n.m.	Infection (7)	n.m.		0
Espinosa 1999	10	1	44 (13–88)	7	3	4	No response (5) Patient choice (1) Device malfunction (4)	Increased impedance (4)	Lead fracture (4)	0
Giulioni 2012	1	0	120	1	0	1	Device malfunction (1)	n.m.	Lead fracture (1)	0
Khurana 2007	2	2	n.m.	2	0	2	Infection (1) Adverse event (1)	Intractable cough (1)	None	0
Landy 1993	2	0	0.5-17	2	0	1	Device malfunction (1) Adverse event (1)	Left vocal cord palsy (1)	Lead fracture (1)	0
Mac Donald 2004	7	n.m.	12 (1–17)	7	0	4	No response (2) Adverse event (1) Infection (2) Device malfunction (2)	Laryngospasm (1) Paresthesia (1)	Lead fracture (1)	0
Ng 2010	8	7	38 (6–108)	7	1	5	Device malfunction (5) Infection (2) Imaging required (1)	Increased seizure frequency (2) Increased impedance (5)	Lead fracture (1)	0
Ortler 2010	9	3	39(13–68)	9	0	0	No response (9) Adverse event (2)	Increased seizure frequency (1)		Vocal cord paresis (1 permanent, 1 temporary)
Rychlicki 2006	3	2	>36	2	1	2	Device malfunction (2) No response (1)	Increased impedance (2)	Lead fracture (2)	Temporary vocal cord paresis (1)
Spitz 2010	1	0	2.5	1	0	1	Adverse event (1)	Pain, dyspnea, phrenic nerve paralysis	Discontinuity insulation	0
Spuck 2010	9	nm	n.m.	7	2	7	Device malfunction (8) Adverse event (1)	Contraction sternocleidomastoid muscle	Lead fracture (8) Lead dislocation (1)	0
Tanganelli 2002	3	n.m.	n.m.	3	0	0	Adverse event (2) Device malfunction (1)	Pain (1) Inflammation (1)	n.m.	0
Tran 2011	1	0	12	1	0	1	Device malfunction (1)	Increased seizure frequency Dysphonia (1)	Traumatic lead fracture (1)	0
Trout 2013	1	1	8	1	0	1	Device malfunction (1)	New seizure type	Lead fracture (1)	0
Vassilyadi 2003	1	1	1	1	0	0	Infection (1)	Abnormal wound (1)	-	Temporary vocal cord paralysis (1)
Waseem 2014	14	n.m.	78 (48–108)	14	0	11	Device malfunction (10) No response (3) Infection (1)	n.m.	Lead fracture (8) Iatrogenic intraoperative fracture (1)	0
Wozniak 2011	3	3	0.5-2	3	0	2	Infection (3)	Infected wound (3)	-	0

*n.m.* not mentioned

## Discussion

VNS is an adjunctive therapy for refractory epilepsy. The first-generation VNS leads (301, 302) have proven to degrade over time, which results in tears in the silicone coating (Fig. 1) that may lead to electrical failure (high impedance) or stimulation related side effects, or even in complete fracture as seen on X-ray examination. Patients that benefit from VNS carrying a first-generation lead are at risk for lead fracture and may require lead revision at some point in their lives. Moreover, with increasing use of VNS for different indications, the demand for removal or replacement will likely increase. With this paper we demonstrate that a complete revision of any VNS device including the leads is feasible, safe, and effective.

In our experience, revision surgery takes about 30 (removal) to 60 (replacement) minutes longer than an initial implantation (which typically takes approximately 45 min), as careful dissection of fibrous scar tissue surrounding the neurovascular bundle under microscopic magnification is needed. Our first few revisions took significantly longer, however, operative time decreases with increasing lead revision experience [1, 9]. A long interval between implantation and revision does not impede complete removal [9, 12], as we have successfully removed leads that had been implanted more than ten years ago. Of note, we have always observed a cleavage plane in between the nerve on the one end and the helices surrounded by multiple layers of scar tissue on the other end. Similarly, a patient’s age does not affect the feasibility of lead revision [19].

The most common adverse event associated with lead revision surgery is vocal cord paresis, which has been reported in five cases including one of our patients (4/131 (3.0 %) in the literature as compared to 1/35 (2.8 %) in our series) [19, 22, 29]. The incidence therefore seems to be slightly higher than after initial implantation, where vocal cord paresis has been reported in approximately 1 % of patients [5]. Vocal cord paresis results from injury to the fibers of the recurrent laryngeal nerve as a result of direct surgical trauma, disruption of the delicate vascularization, and/or secondary inflammation in the nerve’s surroundings, in which case paresis may appear several weeks after revision [22, 29]. In most patients, symptoms subsided completely, even though vocal cord paresis remained in two of them.

Both in our series and in the literature, lead replacement is usually performed because of infection or device malfunction, the former being reported in 3–6 % of patients after initial implantation [5]. Lead salvage by prolonged antibiotic therapy with or without removing the generator may be attempted [31], but persistent infection will necessitate removing all hardware. Lead malfunction usually results from tears in the silicone coating (Fig. 1) or even a complete fracture that occurs either spontaneously or after a trauma. In our series, we observed eight lead failures. In the near future, we expect to see more patients with a first-generation lead (301, 302) requiring lead replacement, however, it is hoped the second-generation leads (303, 304) will prove to be more fatigue-resistant with fever patients requiring revisions, even long term.

Finally, with regard to the efficacy of a replaced VNS system, Dlouhy et al. reported that the replaced VNS system was as effective as the initial one in 15 out of 16 cases they had operated [9]. Waseem et al. reported that nine out of ten patients reported an equal or improved clinical response compared with their initial VNS system, and none of them reported a worse quality of life [30]. The number of anti-epileptic drugs was unaltered in the vast majority of patients [30]. The answers to our questionnaire seem to confirm these findings. However, they are self-reported and therefore subject to a placebo effect. In order to prove that VNS revision including lead replacement does not negatively influence VNS effectiveness, more research including meticulous analysis from long-term neurological follow-up data is needed.

## Conclusions

Our institutional series and overview of the literature confirms that complete removal of all VNS hardware (including the lead and three helical coils) is technically feasible and safe. Although initial results seem promising, further research and longer follow-up are needed to assess whether lead replacement may affect VNS effectiveness. The fact that VNS can be considered fully reversible makes it an even more attractive treatment option.

## Electronic supplementary material

Video 1 Complete removal and reimplantation of the vagus nerve lead and coils in a 9-year-old boy with a VNS type 103 implanted 3 years ago for refractory epilepsy (same patient as in Fig. 1). A few weeks before the operation, the system started malfunctioning with high impedance but with no apparent lead discontinuity. During surgery, degradation of the silicone coating of the lead was observed, and the entire system was replaced. The video shows the left vagus nerve surrounded by coils and scar tissue gently lifted out of the neurovascular bundle by a blue vessel loop. Perineural scar tissue and coils are carefully removed one helix at a time in a cranio-caudal direction using a short scissor with blunt, curved tips. Note the cleavage plane between the nerve (with preserved vascularization) and the helices surrounded by scar tissue. The Gelpi retractor is temporarily removed to tunnel the 303 lead and subsequently repositioned parallel to the neurovascular structures. The coils are carefully positioned around the nerve, and the lead is connected to a new 103 generator. (MP4 171217 kb)

## Abbreviations

VNS: Vagus nerve stimulation
